# Mixed Convection Boundary Layer Flow over a Moving Vertical Flat Plate in an External Fluid Flow with Viscous Dissipation Effect

**DOI:** 10.1371/journal.pone.0060766

**Published:** 2013-04-05

**Authors:** Norfifah Bachok, Anuar Ishak, Ioan Pop

**Affiliations:** 1 Department of Mathematics and Institute for Mathematical Research, Universiti Putra Malaysia, UPM Serdang, Selangor, Malaysia; 2 School of Mathematical Sciences, Faculty of Science and Technology, Universiti Kebangsaan Malaysia, UKM Bangi, Selangor, Malaysia; 3 Faculty of Mathematics, Babeş-Bolyai University, Cluj-Napoca, Romania; University of Catania, Italy

## Abstract

The steady boundary layer flow of a viscous and incompressible fluid over a moving vertical flat plate in an external moving fluid with viscous dissipation is theoretically investigated. Using appropriate similarity variables, the governing system of partial differential equations is transformed into a system of ordinary (similarity) differential equations, which is then solved numerically using a Maple software. Results for the skin friction or shear stress coefficient, local Nusselt number, velocity and temperature profiles are presented for different values of the governing parameters. It is found that the set of the similarity equations has unique solutions, dual solutions or no solutions, depending on the values of the mixed convection parameter, the velocity ratio parameter and the Eckert number. The Eckert number significantly affects the surface shear stress as well as the heat transfer rate at the surface.

## Introduction

Mixed convection flows, or combined forced and free convection flows, arise in many transport processes both naturally and in engineering applications. They play an important role, for example, in atmospheric boundary-layer flows, heat exchangers, solar collectors, nuclear reactors and in electronic equipment. Such processes occur when the effects of buoyancy forces in forced convection or the effects of forced flow in free convection become significant. The interaction of forced and free convection is especially pronounced in situations where the forced flow velocity is low and/or the temperature differences are large. This flow is also a relevant type of flow appearing in many industrial processes, such as manufacture and extraction of polymer and rubber sheets, paper production, wire drawing and glass-fiber production, melt spinning, continuous casting, etc. (Tadmor and Klein [1]). This flow has also many industrial applications such as heat treatment of material traveling between a feed roll and wind-up roll or conveyer belts, extrusion of steel, cooling of a large metallic plate in a bath, liquid films in condensation process and in aerodynamics, etc. As per standard texts books by Bejan [2], Kays and Crawford [3], Bergman et al. [4] and other literatures the free and mixed convection flow occur in atmospheric and oceanic circulation, electronic machinery, heated or cooled enclosures, electronic power supplies, etc. This topic has also many applications such as its influence on operating temperatures of power generating and electronic devices. In addition it should be mentioned that this type of flow plays a great role in thermal manufacturing applications and is important in establishing the temperature distribution within buildings as well as heat losses or heat loads for heating, ventilating and air conditioning systems (see Abraham and Sparrow [5], and Sparrow and Abraham [6]). As it is well-known, the difference between convective heat transfer and forced convection problems is thermodynamic and mathematical, as well, the convective flows being driven by buoyancy effect due to the presence of gravitational acceleration and density variations from one fluid layer to another (Bejan [2]). A considerable amount of research has been reported on this topic (Jaluria [7], Karve and Jaluria [8], etc.). Similarity solutions for moving plates were investigated also by many authors. Among them, Afzal et al. [9], Afzal [10], [11], Fang [12], Fang and Lee [13], Weidman et al. [14], Ishak et al. [15] studied the boundary layer flow on a moving permeable plate parallel to a moving stream and concluded that dual solutions exist if the plate and the free stream move in the opposite directions. The steady mixed convection boundary layer flow on a vertical surface without the effect of viscous dissipation was studied by Dey and Nath [16], while Hieber [17], Schneider [18], Afzal and Hussain [19], Ishak [20] and Ishak et al. [21] studied the mixed convection flow above a heated horizontal surface. Schneider [18] reported that solutions do not exist if the buoyancy parameter is smaller than a certain critical value. Afzal and Hussain [19] reinvestigated this problem and reported the existence of dual solutions in the neighborhood of the separation region.

Different from Dey and Nath [16], the present paper considers the case of a moving plate and the viscous dissipation term in the energy equation is taken into consideration. Like the forced convection, the problem of free convection boundary layer flow near a continuously moving surface has also attracted considerable interest of many authors, such as Ingham [22], because it has many practical applications in manufacturing processes. Ingham [22] showed that the problem of free convection boundary layer flow near a continuously moving vertical plate has non-unique solutions. Merkin [23] probably is the first author who found the existence of dual (non-unique) solutions for mixed convection flow, when he investigated the boundary layer flow in a saturated porous medium. Thereafter, the existence of dual solutions has been pointed out by many researchers, for example, Afzal et al. [9], Weidman et al. [14], Xu and Liao [24], Ishak et al. [25], [26] and Bachok and Ishak [27] when they investigated the boundary layer flow over a moving surface in a parallel stream. It is worth mentioning that the flow and heat transfer characteristics over a moving or stretching surface were analyzed by Chen [28] and Bataller [29]. Recently, a paper by Bachok et al. [30] investigated the flow over a moving surface in a nanofluid. Examples of practical applications of this problem include the aerodynamic extrusion of plastic sheets, the cooling of an infinite metallic plate in a cooling bath, glass blowing, continuous casting spinning fibers, etc. The design of a thermal processing station for moving surfaces requires a knowledge of heat transfer rates and corresponding surface temperature variations (Sparrow and Abraham [6]).

## Problem Formulation

Consider a steady mixed convection boundary layer flow of a viscous and incompressible fluid over a moving vertical flat plate in an external moving fluid. We consider a Cartesian coordinate system 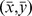 in which the 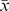-axis is measured along the plate in the upward direction and the 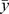-axis is measured in the direction normal to the plate. It is assumed that the velocities of the free stream (or inviscid flow) and the flat plate are 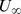 and 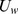, respectively and the viscous dissipation term is 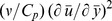 (see Bejan [2]). It is also assumed that the temperature of the plate is 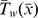, while the uniform temperature of the free stream (inviscid flow) is 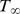. Under these assumptions, the boundary layer equations are (Ingham [22])



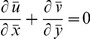
(1)

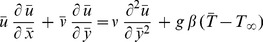
(2)

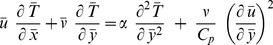
(3)subject to the bounday conditions
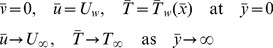
(4)where 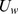 and 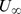 are constants with 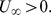 In the above equations, 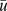 and 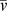 are the fluid velocities in the 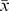 and 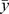 directions, respectively, 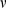 is the kinematic viscosity, 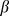 is the thermal expansion coefficient, 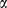 is the thermal diffusivity, 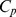 is the specific heat at constant pressure and 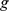 is the acceleration due to gravity. Different from Dey and Nath [16], the present paper considers the case of a moving plate and takes into consideration the viscous dissipation term in the energy equation. The present paper is also different from the paper by Ingham [22], who considered the free convection flow in a quiescent fluid.

We introduce now the following dimensionless variables
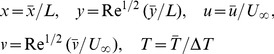
(5)where 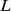 is the characteristic length of the plate, 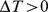 is the characteristic temperature and 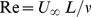 is the Reynolds number. Thus Eqs. (1)–(3) become



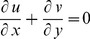
(6)

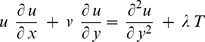
(7)

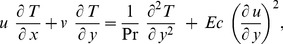
(8)subject to the bounday conditions
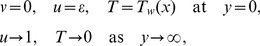
(9)where 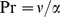 is the Prandtl number, 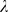 is the mixed convection parameter, 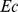 is the Eckert number and 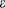 is the velocity ratio parameter, which are defined as [22], [31], [32], [33]

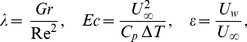
(10)where 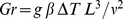 is the Grashof number. It should be mentioned that 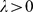 corresponds to assisting flow (heated plate), 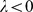 corresponds to opposing flow (cooled plate) and 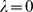 corresponds to forced convection flow.

To obtain similarity solutions of Eqs. (1)–(3), the wall temperature 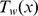 is taken as (see Ingham [22])
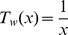
(11)


The idea of “similarity solution” is to simplify the governing equations by reducing the number of independent variables, by a coordinate transformation. The terminology “similarity” is used because, despite the growth of the boundary layer with distance *x* from the leading edge, the velocity as well as the temperature profiles remain geometrically similar. We introduce now the similarity transformation:
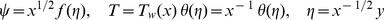
(12)where 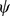 is the stream function defined as 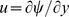 and 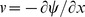 which identically satisfies the continuity equation (6). By this transformation, Eqs. (2) and (3) reduce to the following nonlinear ordinary differential equations:



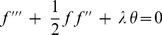
(13)

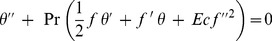
(14)with the boundary conditions



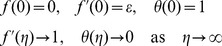
(15)It is worth mentioning that equations (13) and (14) were derived by Ingham [22] but without the viscous dissipation term (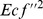) in the energy equation (14). Moreover, the boundary conditions are different, where Ingham [22] considered the free convection flow with 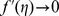 as 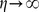.

The physical quantities of interest are the skin friction coefficient 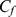
and the local Nusselt number 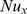, which are respectively defined as
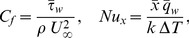
(16)where 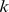 is the thermal conductivity of the fluid, 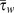 is the surface shear stress and 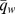 is the surface heat flux, which are given by



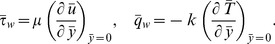
(17)Substituting (5) and (12) into Eqs. (16) and (17), we get
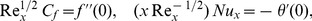
(18)where 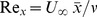 is the local Reynolds number.

## Results and Discussion

Numerical solutions to the nonlinear ordinary (similarity) differential equations (13) and (14) with the boundary conditions (15) were obtained using a shooting method with the help of Maple software. This method is described in details in the recent paper by Aman and Ishak [34]. It was found that these equations have multiple (dual) solutions, which were obtained by setting different initial guesses for the missing values of the skin friction coefficient 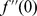 and the local Nusselt number (heat transfer rate) 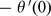, where all profiles satisfy the boundary conditions (15) asymptotically but with different shapes. These two different shapes of profiles produced for a particular value of parameter show that the system of equations (13)–(15) has two solutions. In order to compare the present results with those reported by Dey and Nath [16], we consider also the case when 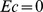 (viscous dissipation is absent) and 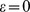 (stationary plate). The quantitative comparison for the values of 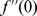 and 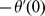 is shown in Table 1, and it is found that they are in a very good agreement. Moreover, the values of 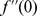 and 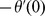 for 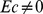 and 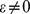 are also included in Table 1 for future references.

**Table 1:** Values of 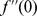 and 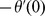 for different values of 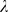, 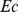 and 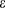 when 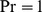.

*λ*	*Ec*	*ε*	Dey and Nath [16]		Present work	
			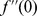	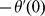	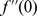	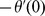
−0.1	0	−0.1			0.307066	0.595873
					[−0.103876]	[0.319708]
		0	0.350971	0.760608	0.350986	0.760823
					[−0.158162]	[0.273451]
		0.1			0.380260	0.973171
					[−0.202513]	[0.217419]
	0.5	−0.1			0.330728	0.602316
					[−0.105064]	[0.312167]
		0			0.373730	0.785243
					[−0.159043]	[0.262877]
		0.1			0.403493	1.024008
					[−0.203813]	[0.201039]
0.1	0	−0.1			0.357351	0.509747
		0	0.332920	0.593633	0.332920	0.593633
		0.1			0.302131	0.691214
	0.5	−0.1			0.344851	0.483145
		0			0.321280	0.577087
		0.1			0.287885	0.696999

• [ ] second solution.

**Figure 1:**
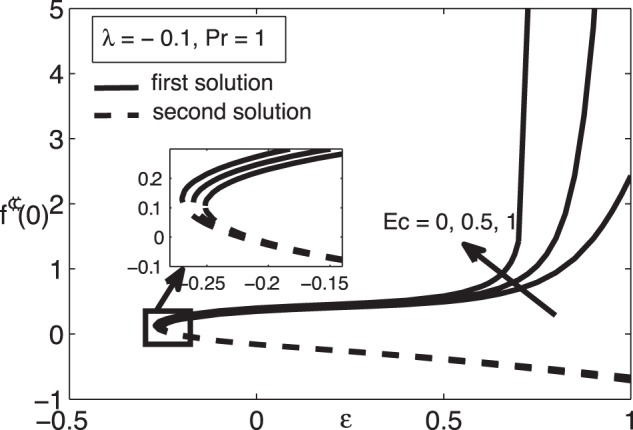
Skin friction coefficient 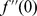 for different values of 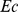 when 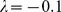 and 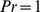.

**Figure 2:**
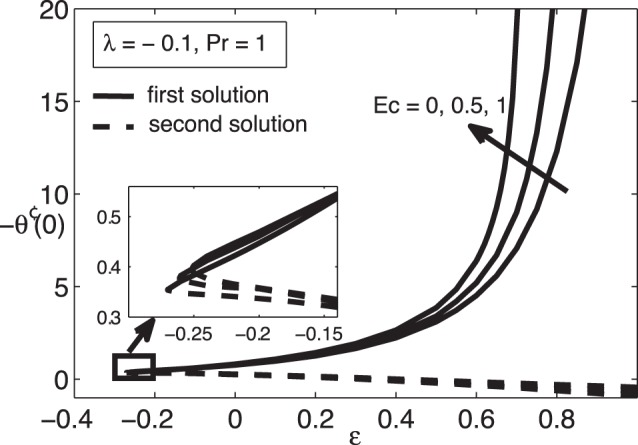
Variation of 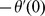 for different values of 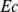 when 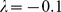 and 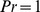.

**Figure 3:**
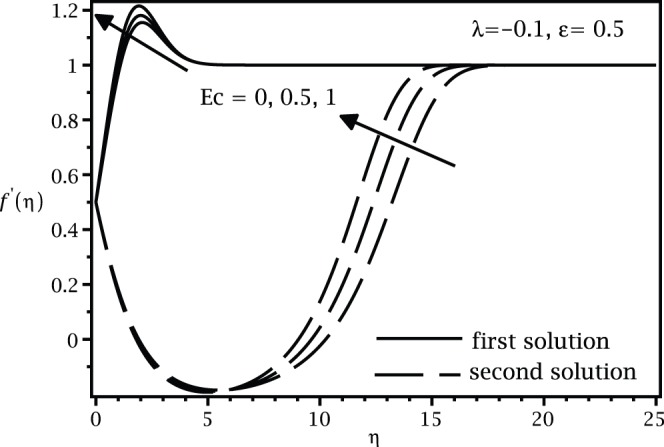
Velocity profiles 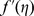 for different values of 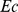 when 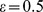, 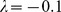 and 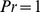.

**Figure 4:**
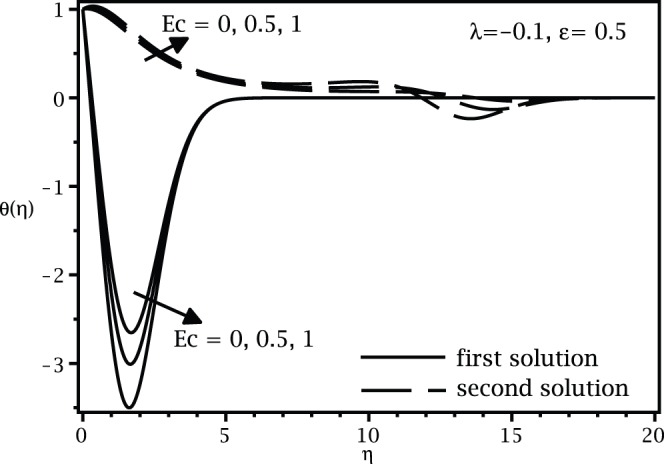
Temperature profiles 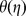 for various values of 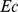 when 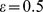, 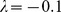 and 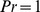.

**Figure 5:**
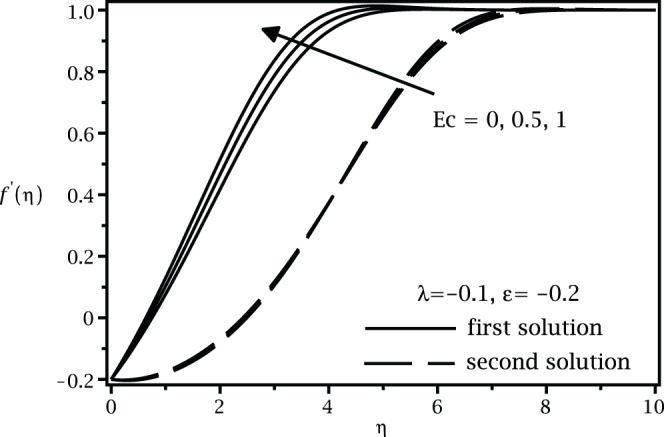
Velocity profiles 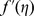 for different values of 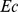 when 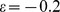, 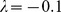 and 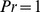.

**Figure 6:**
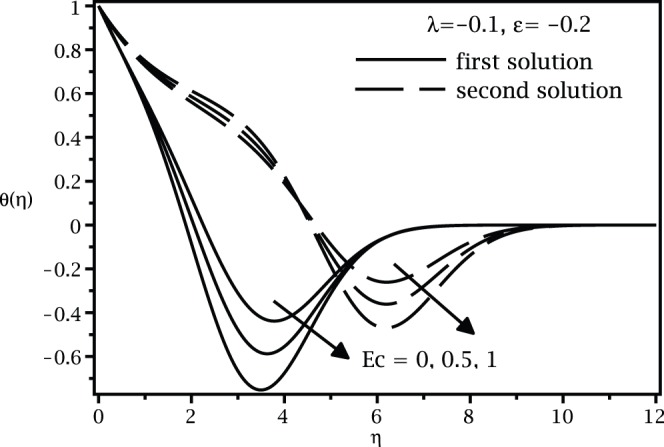
Temperature profiles 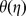 for different values of 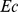 when 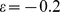, 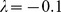 and 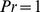.

Figs. 1 and 2 present the variations of 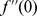 and 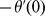 as a function of 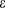 for 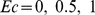 when 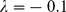 (opposing flow) and 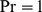, while the samples of the respective velocity and temperature profiles are given in Figs. 3, 4 5, and 6. We found that there are two (dual) solutions, a first (upper branch) solution and a second (lower branch) solution for certain range of 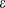. We identify the first (upper branch) and second (lower branch) solutions by how they appear in Figs. 1 and 2, i.e. the first solution has higher values of 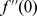 and 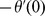 than the second solution, for a given 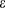. For negative values of 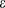, there is a critical value 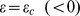 as presented in Table 2, for which the solution of equations (13) and (14) with the boundary conditions (15) exists. There is no solution of these boundary layer equations for 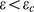. In this case, the full Navier-Stokes and energy equations should be solved in the region 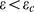 . As can be seen from Figs. 1 and 2, the Eckert number 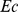 significantly affects the skin friction coefficient 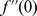 as well as the heat transfer rate at the surface 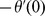. Table 2 shows that the value of 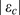 increases with the increase of the Eckert number 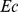. Thus the Eckert number 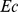 increases the range of 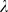 for which the solution exists. Figs. 3, 4, 5, and 6 show that the boundary conditions (15) as 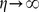 are asymmptotically satisfied, which support the validity of the present results, besides supporting the dual nature of the solution to the boundary-value problem (13)–(15).

**Table 2:** Values of 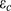 and 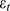 for several values of 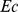 and 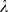 when 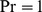.

*λ*	*Ec*	*ε_c_*	*ε_t_*
−0.1	0	−0.2513	
	0.5	−0.2607	
	1	−0.2701	
0.1	0	−0.5516	1.1992
	0.5	−0.5740	1.3130
	1	−0.5998	1.4081

**Figure 7:**
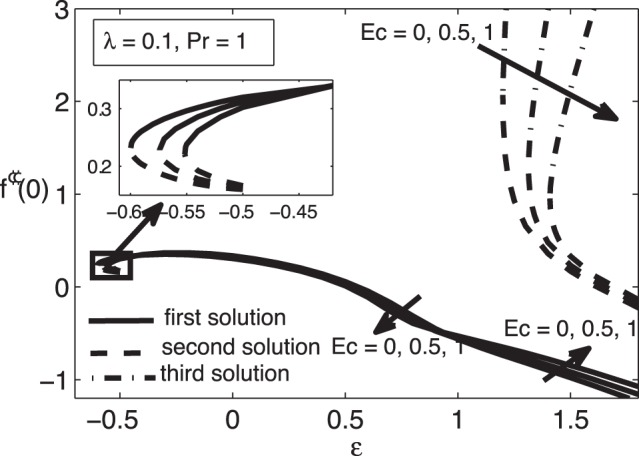
Skin friction coefficient 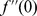 for different values of 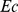 when 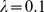 and 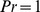.

**Figure 8:**
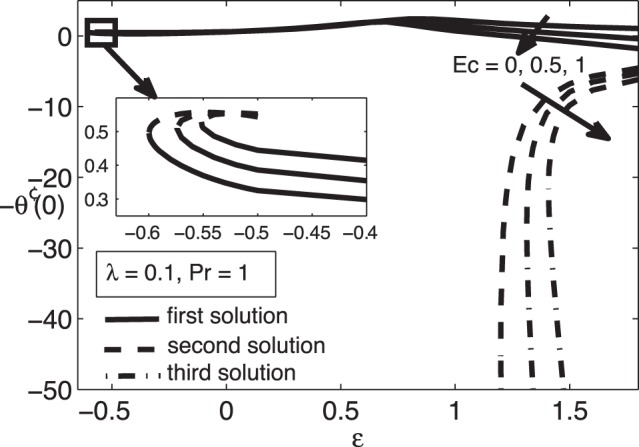
Variation of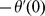 for different values of 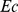 when 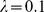 and 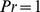.

**Figure 9:**
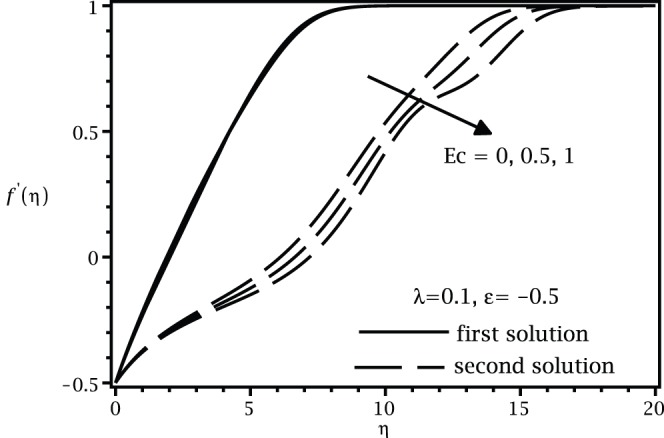
Velocity profiles 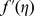 for different values of 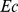 when 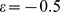, 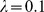 and 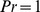.

**Figure 10:**
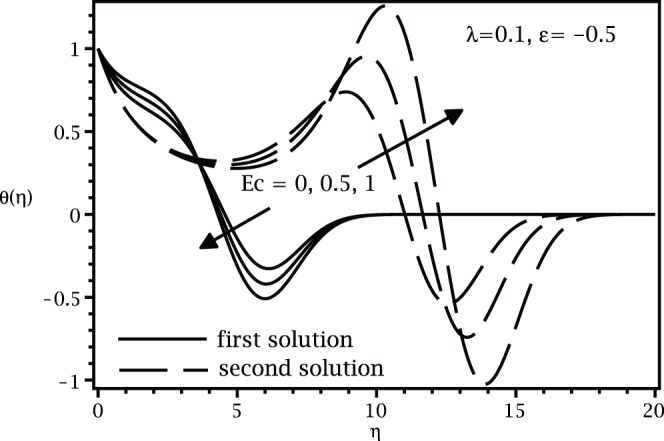
Temperature profiles 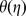 for different values of 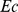 when 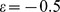, 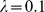 and 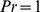.

**Figure 11:**
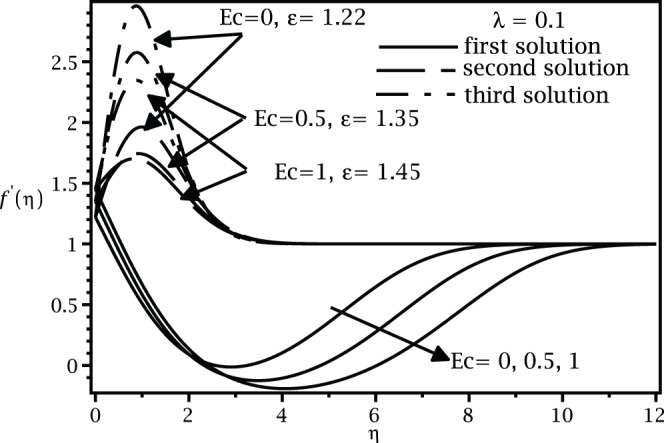
Velocity profiles 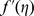 for different values of 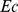 and 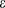 when 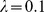 and 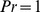.

**Figure 12:**
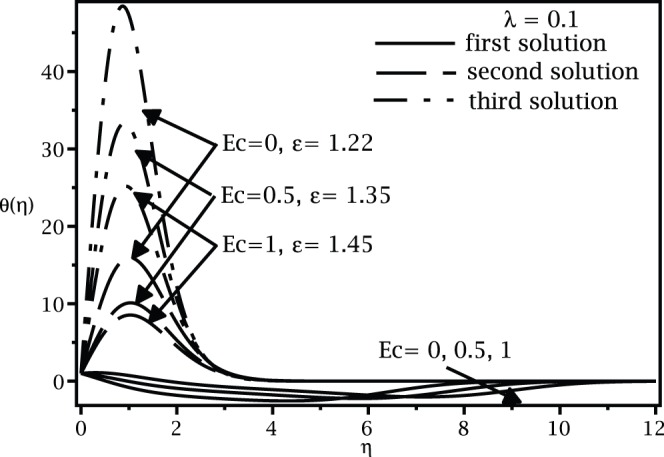
Temperature profiles 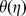 for different values of 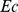 and 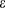 when 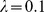 and 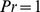.

For positive values of the mixed convection parameter 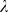 (assisting flow), Figs. 7 and 8 show the variation of 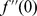 and 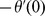 as a function of 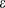 for 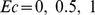 when 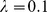 and 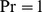, while the samples of the respective velocity and temperature profiles are given in Figs. 9, 10, 11, and 12. It is seen that there are regions of three solutions for 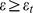, unique solutions for 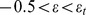, dual solutions for 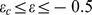 and no solutions for 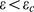, where 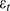 and 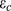 are the critical values of 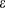 as presented in Table 2. It should be mentioned that such a behaviour near the turning point as shown in Fig. 7 has also been depicted by Weidman et al. [14] for the case of forced convection boundary layer flow over moving surfaces (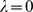) with suction effect.

For the dual solutions, we expect that the upper branch solution is stable and physically realizable while the lower branch solution is not. The procedure for showing this has been described by Merkin [23], Weidman et al. [14], Postelnicu and Pop [35], and very recently by Rosca and Pop [36]. It is worth mentioning to this end that this type of multiple (dual) solutions is essentially a backward flow, and it shows a physical phenomenon quite distinct from the flow with no dual solutions, being important in many practical problems (Fang et al. [37]).

### Conclusions

We have numerically studied the mixed convection boundary-layer flow over a moving vertical flat plate in the presence of an external flow. Using appropriate similarity variables, the governing system of partial differential equations is transformed into a system of ordinary differential equations. This system is then solved numerically using a shooting method. Results for the skin friction coefficient, local Nusselt number, velocity profiles as well as temperature profiles are presented for different values of the governing parameters. It is found that the set of similarity equations has three solutions, dual solutions, unique solution or no solution, depending on the value of the mixed convection parameter and the velocity ratio parameter. The Eckert number significantly affects the surface shear stress as well as the heat transfer rate at the surface.
